# Dynamic Diastereomerism on Chiral Surfaces

**DOI:** 10.1021/acs.jpcc.2c06351

**Published:** 2022-12-30

**Authors:** Sabine C. Matysik, David J. Wales, Stephen J. Jenkins

**Affiliations:** Yusuf Hamied Department of Chemistry, University of Cambridge, Cambridge CB2 1EW, U.K.

## Abstract

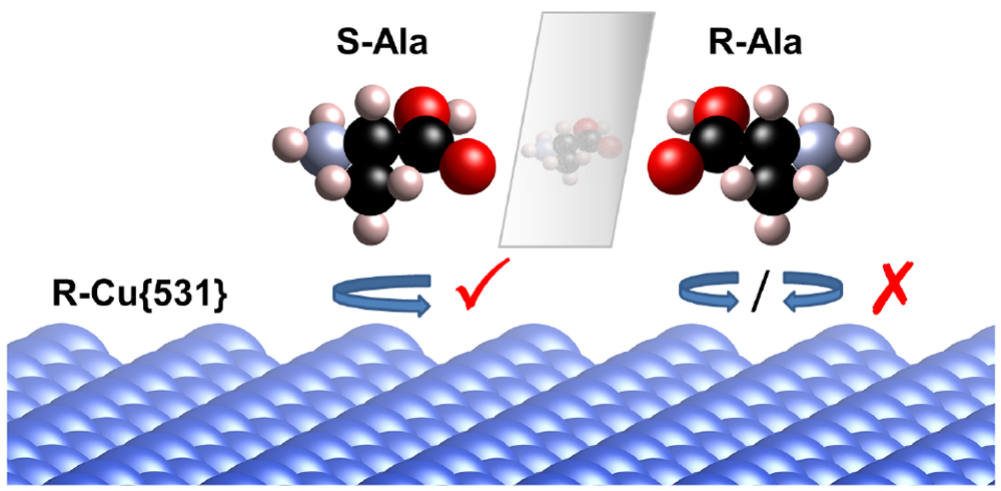

Adsorption of chiral molecules on chiral surfaces implies diastereomerism, evident in the adoption of distinct adsorption geometries. We show here that this diastereomerism produces a signature in the motion of chiral molecules desorbing from a chiral surface. The rotations of *S*- and *R*-alanine molecules are analyzed upon desorption from *R*-Cu{531} using first-principles molecular dynamics simulations. *S*-Ala molecules exhibit a larger angular momentum, with a clear preference for one rotational sense, whereas no such preference is observed for *R*-Ala molecules upon desorption from this surface. These trends would be reversed for desorption from the *S*-Cu{531} surface. Possible applications include chiral separation techniques and enantiospecific sensors.

## Introduction

A chiral object can exist in two enantiomeric forms that are mirror images. When two such chiral objects interact, or when two stereocenters are present in the same system (e.g., the same molecule, such as tartaric acid), diastereomeric forms arise in addition to the two enantiomers. Diastereomers (e.g., *R*,*S*-tartaric acid and *R*,*R*-tartaric acid) are not related through mirror symmetry, but constitute nonsuperimposable stereoisomers. While enantiomers and enantiomeric behavior can only be distinguished by their interaction with a chiral environment, diastereomeric systems reveal themselves through much more easily discernible differences in their properties. Precisely how these differences will manifest themselves is, however, not easy to predict.

A system with two chiral entities arises when a chiral adsorbate interacts with a chiral surface. The adsorption of amino acids has become an active area of research within the field of surface chirality, due to the close connection with biochemically and pharmaceutically important molecules.^1^ Studying amino acid adsorption on chiral surfaces has revealed various phenomena, including diastereomeric differences in local adsorption geometry, long-range order, and decomposition kinetics.^2−7^ Alanine, in particular, as the smallest chiral amino acid, has attracted a lot of interest, and it has been studied with a variety of experimental and computational techniques.^2,8−11^

The adsorption of alanine on chiral and achiral copper surfaces provides a rich hierarchy of chirality on several levels. On a molecular level, alaninate adsorbs with a three-point interaction through both its carboxylate oxygens and the nitrogen atom of its amino group. This μ_3_ interaction is often called the (triangular) footprint. On Cu{110}, this footprint is itself chiral, even though the surface is achiral. So, in addition to the molecular chirality of the amino acid molecule itself, a chiral local adsorption structure can form. On chiral surfaces such as Cu{531}, diastereomeric local adsorption geometries are therefore possible and indeed observed.^4,9^ At a third level, chiral overlayers with long-range order were observed on Cu{111},^12^ Cu{110},^13^ and Cu{311}.^2^

We have previously demonstrated enantiomeric effects in the desorption of formic acid (an achiral molecule) from a chiral copper surface.^14^ In the present study, we investigate how diastereomeric combinations of *S*- and *R*-alanine with *R*-Cu{531} influence the rotation of alanine molecules upon desorption, which is a dynamical manifestation of the underlying diastereomerism. To the best of our knowledge, no such dynamic diastereomerism has been reported before.

## Methods

We employ our previous methodology for extracting reactive trajectories that are connected to an adsorption and dissociation event, using only desorption trajectories that pass through the transition state for dissociation on the copper surface.^14−18^ In short, the adsorption geometries of alanine and alaninate on Cu{531} and Cu{110} were used as starting points to locate the transition state necessary for initializing the MD simulations. All DFT calculations were performed with the CASTEP code (Version 18.1)^19^ using the Perdew–Burke–Ernzerhof exchange-correlation functional,^20^ ultrasoft pseudopotentials,^21^ a cutoff energy of 500 eV for the plane-wave basis and the Tkatchenko–Scheffler dispersion force correction scheme.^22^ The transition state search was performed through preoptimization using the LST-QST^23^ algorithm, as implemented in CASTEP, followed by accurate refinement using hybrid eigenvector-following^24−26^ with the OPTIM code until the RMS gradient fell below 0.001 eV Å^–1^. Normal mode frequencies were obtained by finite-displacement calculations. Based on the equipartition theorem for the transition state, the same amount of kinetic energy for each trajectory was distributed equally among these modes, i.e. *k*_*B*_*T* in each normal mode, apart from the reaction coordinate, which, due to its single quadratic degree of freedom, was given only *k*_*B*_*T*/2 (*T* = 300 K). Each mode was assigned a randomly chosen phase, and thus different initial velocities for MD trajectories with the same amount of assigned kinetic energy were generated. MD simulations were performed using an *NVE* ensemble (constant number *N* of particles, volume *V*, and total energy *E*) and a time step of 0.5 fs. A total of 48 trajectories were computed per surface, of duration 200 fs each. Along with the *k*_*B*_*T* kinetic energy assignment to the normal modes, we note that full desorption from the surface was only achieved if another 1.8 eV of translational energy parallel to the surface normal (*z*-axis of the supercell) was assigned to the molecular center of mass. In this case, we find the mean velocity of our desorbing molecules to be around 800 m s^–1^ (see Section 3 of the Supporting Information). We thereby focus, in effect, on a subset of molecules from the high-energy tail of the Maxwell–Boltzmann energy distribution, which are most likely to fully desorb and produce an experimental signature. For more details of the methodology, we refer to our previous work.^14^

## Results and Discussion

**Figure 1:**
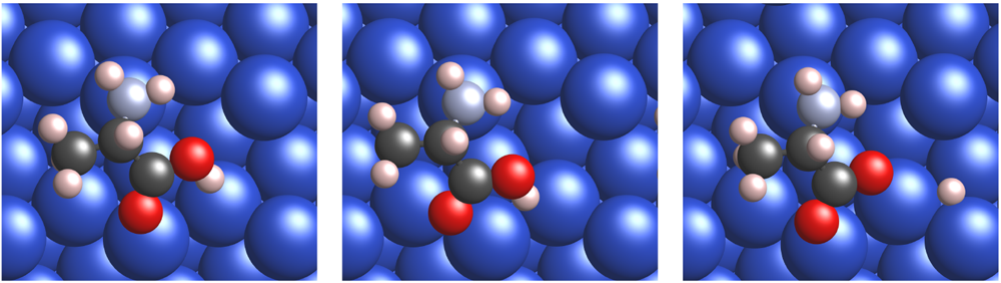
*S*-Alanine dissociation on *R*-Cu{531}. Atoms shown in red (oxygen), dark gray (carbon), light blue (nitrogen), off-white (hydrogen), and dark blue (copper). (Left) Intact alanine, (middle) transition state, and (right) alaninate and hydrogen after dissociation.

**Figure 2:**
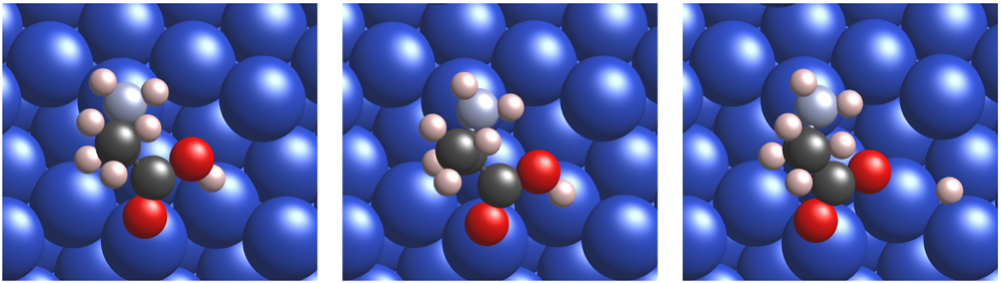
*R*-Alanine dissociation on *R*-Cu{531}. (Left) Intact alanine, (middle) transition state, and (right) alaninate and hydrogen after dissociation.

The adsorption geometries of intact alanine (left panel of Figure 1), alaninate (right panel of Figure 1), and the corresponding transition state (middle panel of Figure 1) for deprotonation/protonation of the carboxylic/carboxylate group are shown in Figure 1 for *S*-Ala and similarly in the left, right and middle panels of Figure 2, respectively, for *R*-Ala on the {311} microfacet of Cu{531}. The {110} and {311} microfacet adsorption sites have previously been shown to be the two experimentally observable adsorption sites for the μ_3_-adsorption of alaninate on *R*-Cu{531}.^9^ Note that *S*-Ala on *S*-Cu{531} would be the mirror image of *R*-Ala on *R*-Cu{531}, while *R*-Ala on *S*-Cu{531} would be the mirror image of *S*-Ala on *R*-Cu{531}, so neither of these combinations need be computed explicitly. We found that the {311} adsorption site is more energetically favorable than the {110} site for both alanine and alaninate (see Section 1 of the Supporting Information), and hence, we focus on the dissociative adsorption at the {311} site.

**Figure 3:**
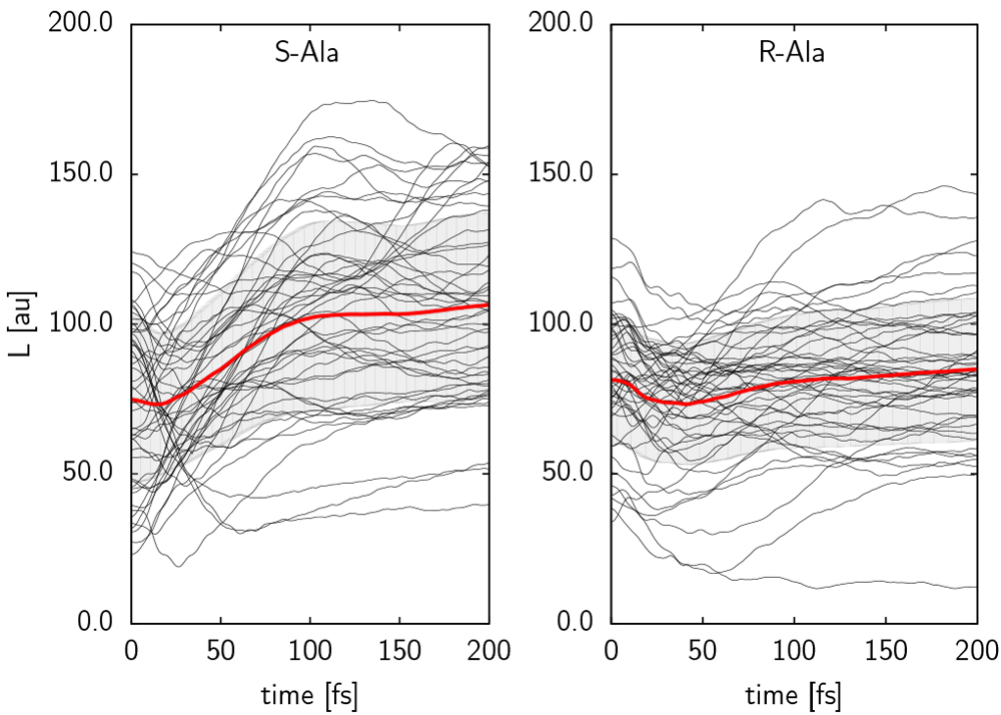
Time evolution of the magnitude of the angular momentum, *L*, of (left) *S*-Ala and (right) *R*-Ala on Cu{531}. Red continuous lines indicate the mean value for the whole ensemble of trajectories desorbing from the surface; an error range of one standard deviation is indicated in gray.

The MD trajectories started from the transition states were then analyzed, focusing on their rotational characteristics. The magnitude of the angular momentum vector, **L**, for each trajectory, together with the mean for each set is shown in Figure 3 for both *S*- and *R*-Ala desorbing from Cu{531}. The values are given in atomic units (au; 1 au = *ℏ*). For *S*-Ala an increase of *L* (= |**L**|) by 42% is seen during the simulation time, while *L* remains almost constant (3% increase) for *R*-Ala. Most of the increase in *L* for *S*-Ala occurs during the first 100 fs of the simulation, which coincides with the time for an average distance of 4 Å between the reassociating hydrogen and the topmost copper atom to be achieved.

**Figure 4:**
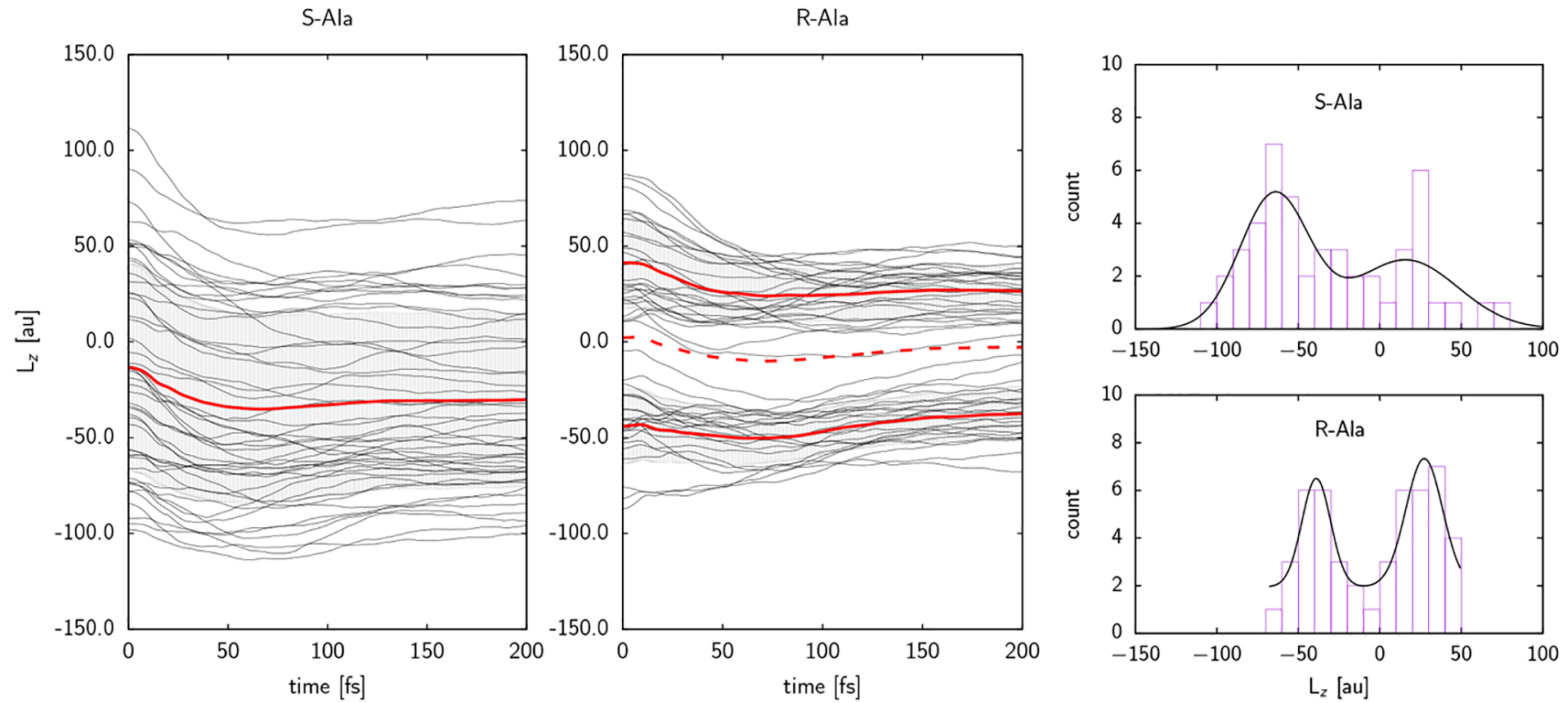
Characteristics of the *z*-component of the angular momentum, *L*_*z*_. (Left) Time evolution of *L*_*z*_ of *S*-Ala and (middle) *R*-Ala on *R*-Cu{531}. Red continuous lines indicate the mean value for (left) the whole ensemble of trajectories of *S*-Ala desorbing from *R*-Cu{531} and (middle) the mean values for each subpopulation of *R*-Ala trajectories, while an error range of one standard deviation is indicated in gray. The red dashed line indicates the mean value for the whole ensemble of *R*-Ala trajectories. (right) Histogram of the distribution of values of *L*_*z*_ at *t* = 200 fs for (top) *S*-Ala and (bottom) *R*-Ala having desorbed from *R*-Cu{531}. Gaussian fits to the data (Cramér-von Mises p-values greater than 0.99) have been overlaid on the right-hand panels (see Section 2 of the Supporting Information for details).

Turning to the *z*-component of the angular momentum vector, *L*_*z*_, the ensemble of *S*-Ala trajectories exhibits a robustly negative value (left panel of Figure 4). *L*_*z*_ is the only component of **L** that is unique to a chiral system because of the pseudovector nature of the angular momentum. When a pseudovector is reflected the sign of the component parallel to the mirror plane changes, while the signs of the components perpendicular to the mirror plane remain constant. Thus, when both a surface-bound transition state and its mirror image give rise to an angular momentum, its *z*-component (which will be parallel to the mirror plane) will cancel when averaging over both transition states. On a chiral surface, however, no such mirror-image transition state can exist and any observed *L*_*z*_ cannot be canceled out. Our previous results show that surface chirality can induce a directed angular momentum with a nonzero *L*_*z*_ value in desorbing achiral molecules.^14^ For chiral molecules, a similar behavior seemed plausible, potentially with a difference in magnitude of the observed effect. This possibility inspired the present investigation. The effect observed for *S*-Ala is therefore in line with our expectations for a chiral system. The ensemble of *R*-Ala trajectories does not, however, exhibit the expected behavior (middle panel of Figure 4). The *L*_*z*_ values of each trajectory fall into one of two subpopulations, one positive and one negative, with an overall average of approximately zero. This behavior is very similar to that observed previously for entirely achiral systems (achiral adsorbate and achiral surface), e.g. formic acid on Cu{110}.^14^ The expected chiral effect on the preferred rotations of the desorbing molecules seems to have been canceled out by a “mismatch” between the chirality of the adsorbate and that of the surface. To rule out any unexpected rotational behavior of a chiral molecule desorbing from an achiral surface, we also calculated 48 desorption trajectories of *R*-Ala on Cu{110}. The results are consistent with our previous findings for chiral systems with only one chiral component, see Section 4 of the Supporting Information.

To further test the statistical robustness of this difference between *S*-Ala and *R*-Ala, the right panel of Figure 4 shows a histogram plot of the distribution of *L*_*z*_ values. It is evident that the values for *R*-Ala obey a well-resolved and rather symmetric bimodal distribution, while those for *S*-Ala obey a highly asymmetric and barely resolved bimodal distribution. The observed directionality of the angular momentum vectors for *S*-Ala is further strengthened by the narrowing of the spread of individual vectors around their ensemble mean, which decreases from an average angle between individual angular momentum vectors and the ensemble mean of 66° at 20 fs, to 45° at 60 fs, to 37° at the end of the simulation. For *R*-Ala this decrease is much less pronounced (71° at 20 fs, 60° at 60 fs, and 56° at 200 fs). Further information on the vector distribution can be found in Section 5 of the Supporting Information.

**Figure 5:**
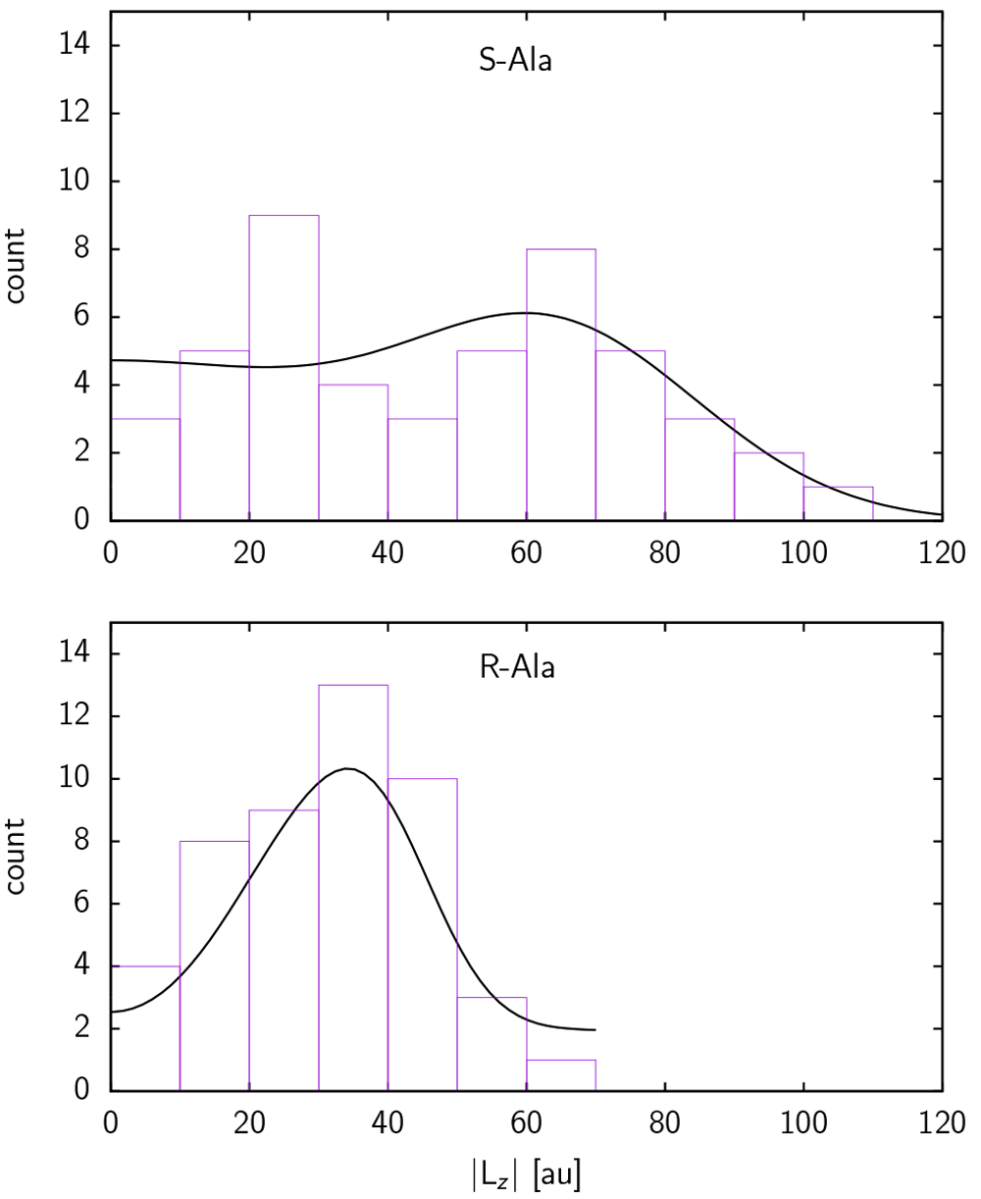
Histogram of the distribution of the absolute values for *L*_*z*_, |*L*_*z*_| at *t* = 200 fs for (top) *S*-Ala and (bottom) *R*-Ala having desorbed from *R*-Cu{531}. Gaussian fits from Figure 4 have been summed over positive and negative values of *L*_*z*_ and overlaid upon the histograms.

The plots in Figure 4 imply knowledge about the sense of rotation, indicated by the sign of *L*_*z*_, and an experimental verification would thus rely on the ability to detect or selectively produce either rotational sense, similar to what our previous findings on achiral molecules would necessitate.^14^ The rotational behavior of *S*-Ala and *R*-Ala, however, also leads to different signatures in the distribution of the absolute value of *L*_*z*_, as shown in Figure 5. Evidently, |*L*_*z*_| of *S*-Ala obeys a more bimodal type of distribution, with two maxima around 20 au and 70 au, while |*L*_*z*_| of *R*-Ala exhibits a nearly unimodal distribution, with only one clear maximum around 35 au. Since diastereomeric effects cannot spontaneously arise without a diastereomeric cause, we are logically compelled to conclude that the distinguishable features of the two angular momentum distributions arise directly from the diastereomerism of the two different transition states. What is remarkable, and the main conclusion of the present work, is that this link is not entirely obscured by the randomized phases we apply to our initial velocities. Diastereomerism, therefore, can be expected to be observable in practice, despite the stochastic nature of real-world desorption (or, in time-reversal, adsorption) processes. Due to these distinctive dynamic distributions, the diastereomerism of the rotations of *S*-Ala and *R*-Ala desorbing from *R*-Cu{531} would be amenable to experimental verification. The experiment would not need to distinguish between clockwise and anticlockwise rotating molecules, requiring only the ability to detect rotational speed. We note that control over rotational states in molecular beams can be achieved via laser excitation but that selectivity between the two senses of rotation is much more challenging, as discussed by Fleischer et al.^27^

## Conclusion

We conclude that *S*- and *R*-Ala show very different rotational properties upon desorption from *R*-Cu{531}. *S*-Ala exhibits a larger and more directed angular momentum with a clear preference for one rotational sense, whereas *R*-Ala shows no significant increase in angular momentum upon desorption, and no preference for a certain sense of rotation. This notably different behavior is a clear manifestation of the underlying diastereomerism of the two systems, which is not limited to adsorption geometries and energetics, but correlates with the motion of the molecules after they have fully desorbed. Assuming the principle of microscopic reversibility, this behavior upon desorption translates into a preferred rotational direction for adsorption events of *S*-Ala on *R*-Cu{531} but no such preference for adsorption events of *R*-Ala. These preferences would be reversed for adsorption on *S*-Cu{531}. Additionally, this dynamic diastereomerism also leads to different signatures in the angular momentum distribution, even without knowledge of the rotational sense. This result substantially simplifies experimental verification and utilization of these effects, and our result should therefore be able to inform customized supersonic beam experiments. As the ability to distinguish between enantiomers is often critical in, for instance, the pharmaceutical industry, such diastereomeric effects may have important implications for the application of chiral surfaces in chiral recognition and chiral resolution processes. Dynamic effects in the course of desorption are among the most promising avenues for achieving chiral amplification through surface interactions,^1^ and the rotational example described here deserves further analysis.

## Data Availability

The data that support the findings of this study are openly available in the Apollo repository at 10.17863/CAM.82942

## Supporting Information Available

Additional details on the geometry optimization process for alanine on Cu{531}, the statistical evaluation of the MD results and the desorption velocity of alanine molecules from Cu{531} are given. Furthermore, the desorption of *R*-Ala molecules from Cu{110} is briefly summarized and the principal moments of inertia alongside the angular momentum vectors are shown for *R*- and *S*-Ala on Cu{531} (PDF)

## References

[ref1] Jenkins S. J. Chirality at Solid Surfaces; John Wiley & Sons, Ltd.: Chichester, U.K., 2018.

[ref2] Clegg M. L.; Morales De La Garza L.; Karakatsani S.; King D. A.; Driver S. M. Chirality in amino acid overlayers on Cu surfaces. Top. Catal. 2011, 54, 1429–1444. 10.1007/s11244-011-9758-y.

[ref3] Eralp T.; Ievins A.; Shavorskiy A.; Jenkins S. J.; Held G. The importance of attractive three-point interaction in enantioselective surface chemistry: Stereospecific adsorption of serine on the intrinsically chiral Cu{531} surface. J. Am. Chem. Soc. 2012, 134, 9615–9621. 10.1021/ja210499m. 22582880

[ref4] Song H. S.; Han J. W. Tuning the Surface Chemistry of Chiral Cu(531)S for Enhanced Enantiospecific Adsorption of Amino Acids. J. Phys. Chem. C 2015, 119, 15195–15203. 10.1021/acs.jpcc.5b02695.

[ref5] Yun Y.; Gellman A. J. Enantiospecific Adsorption of Amino Acids on Naturally Chiral Cu{3,1,17}R&S Surfaces. Langmuir 2015, 31, 6055–6063. 10.1021/acs.langmuir.5b00707. 25933641

[ref6] Gladys M. J.; O’Donnell K.; Tadich A.; Yook H.; Han J. W.; Thomsen L. Enantiospecific Adsorption and Decomposition of Cysteine Enantiomers on the Chiral Cu{421} R Surface. J. Phys. Chem. C 2019, 123, 20829–20837. 10.1021/acs.jpcc.9b03373.

[ref7] Dutta S.; Gellman A. J. Enantiospecific equilibrium adsorption and chemistry of d-/l-proline mixtures on chiral and achiral Cu surfaces. Chirality 2020, 32, 200–214. 10.1002/chir.23153. 31762092

[ref8] Jones G.; Jones L. B.; Thibault-Starzyk F.; Seddon E. A.; Raval R.; Jenkins S. J.; Held G. The local adsorption geometry and electronic structure of alanine on Cu{1 1 0}. Surf. Sci. 2006, 600, 1924–1935. 10.1016/j.susc.2006.02.033.

[ref9] Gladys M. J.; Stevens A. V.; Scott N. R.; Jones G.; Batchelor D.; Held G. Enantiospecific adsorption of alanine on the chiral Cu{531} surface. J. Phys. Chem. C 2007, 111, 8331–8336. 10.1021/jp070621f.

[ref10] Yun Y.; Wei D.; Sholl D. S.; Gellman A. J. Equilibrium adsorption of d - And l -Alanine mixtures on naturally chiral Cu{3,1,17}R&S surfaces. J. Phys. Chem. C 2014, 118, 14957–14966. 10.1021/jp503796u.

[ref11] Gladys M. J.; Han J. W.; Pedersen T. S.; Tadich A.; O’Donnell K. M.; Thomsen L. Adsorption differences between low coverage enantiomers of alanine on the chiral Cu{421}R surface. Phys. Chem. Chem. Phys. 2017, 19, 13562–13570. 10.1039/C7CP01844D. 28513743

[ref12] Baldanza S.; Cornish A.; Nicklin R. E. J.; Zheleva Z. V.; Held G. Surface chemistry of alanine on Cu{111}: Adsorption geometry and temperature dependence. Surf. Sci. 2014, 629, 114–122. 10.1016/j.susc.2014.04.016.

[ref13] Rankin R. B.; Sholl D. S. Structure of enantiopure and racemic alanine adlayers on Cu(1 1 0). Surf. Sci. 2005, 574, L1–L8. 10.1016/j.susc.2004.10.025. 16853135

[ref14] Matysik S. C.; Wales D. J.; Jenkins S. J. Surface Chirality Influences Molecular Rotation upon Desorption. Phys. Rev. Lett. 2021, 126, 166101 10.1103/PhysRevLett.126.166101. 33961485

[ref15] Sacchi M.; Wales D. J.; Jenkins S. J. Mode-Specific Chemisorption of CH4 on Pt{110}-(1 × 2) Explored by First-Principles Molecular Dynamics. J. Phys. Chem. C 2011, 115, 21832–21842. 10.1021/jp207746q.

[ref16] Sacchi M.; Wales D. J.; Jenkins S. J. Mode-specificity and transition state-specific energy redistribution in the chemisorption of CH4 on Ni{100}. Phys. Chem. Chem. Phys. 2012, 14, 15879 10.1039/c2cp42345f. 23092950

[ref17] Sacchi M.; Wales D. J.; Jenkins S. J. Bond-selective energy redistribution in the chemisorption of CH3D and CD3H on Pt{110}-(1 × 2): A first-principles molecular dynamics study. Comput. Theor. Chem. 2012, 990, 144–151. 10.1016/j.comptc.2011.11.048.

[ref18] Matysik S. C.; Wales D. J.; Jenkins S. J. Rotational Dynamics of Desorption: Methane and Ethane at Stepped and Kinked Platinum Surfaces. J. Phys. Chem. C 2021, 125, 27938–27948. 10.1021/acs.jpcc.1c09120.

[ref19] Clark S. J.; Segall M. D.; Pickard C. J.; Hasnip P. J.; Probert M. I. J.; Refson K.; Payne M. C. First principles methods using CASTEP. Zeitschrift für Krist. - Cryst. Mater. 2005, 220, 567–570. 10.1524/zkri.220.5.567.65075.

[ref20] Perdew J. P.; Burke K.; Ernzerhof M. Generalized Gradient Approximation Made Simple. Phys. Rev. Lett. 1996, 77, 3865–3868. 10.1103/PhysRevLett.77.3865. 10062328

[ref21] Vanderbilt D. Soft self-consistent pseudopotentials in a generalized eigenvalue formalism. Phys. Rev. B 1990, 41, 7892–7895. 10.1103/PhysRevB.41.7892. 9993096

[ref22] Tkatchenko A.; Scheffler M. Accurate Molecular Van Der Waals Interactions from Ground-State Electron Density and Free-Atom Reference Data. Phys. Rev. Lett. 2009, 102, 073005 10.1103/PhysRevLett.102.073005. 19257665

[ref23] Govind N.; Petersen M.; Fitzgerald G.; King-Smith D.; Andzelm J. A generalized synchronous transit method for transition state location. Comput. Mater. Sci. 2003, 28, 250–258. 10.1016/S0927-0256(03)00111-3.

[ref24] Munro L. J.; Wales D. J. Defect migration in crystalline silicon. Phys. Rev. B 1999, 59, 3969–3980. 10.1103/PhysRevB.59.3969.

[ref25] Henkelman G.; Jónsson H. A dimer method for finding saddle points on high dimensional potential surfaces using only first derivatives. J. Chem. Phys. 1999, 111, 7010–7022. 10.1063/1.480097.

[ref26] Kumeda Y.; Wales D. J.; Munro L. J. Transition states and rearrangement mechanisms from hybrid eigenvector-following and density functional theory. Chem. Phys. Lett. 2001, 341, 185–194. 10.1016/S0009-2614(01)00334-7.

[ref27] Fleischer S.; Khodorkovsky Y.; Prior Y.; Sh Averbukh I. Controlling the sense of molecular rotation. New J. Phys. 2009, 11, 105039 10.1088/1367-2630/11/10/105039.

