# Supplementary material for: Dynamic Diastereomerism on Chiral Surfaces
Source: J Phys Chem C Nanomater Interfaces. 2022 Dec 30;127(1):229–33. doi: 10.1021/acs.jpcc.2c06351 (PMC9841561; doi:10.1021/acs.jpcc.2c06351)
Supplement: Supplementary file 1 — jp2c06351_si_001.pdf [file jp2c06351_si_001.pdf]

Supporting Information to: Dynamic Diastereomerism on Chiral Surfaces

Sabine C. Matysik, David J. Wales, Stephen J. Jenkins

1 Geometry Optimisation of Alanine on Cu{531}

Table 1: Adsorption energies in eV of intact S- and R-alanine, the corresponding transition state (TS), and S- and R-alaninate adsorbed on the two possible microfacets of Cu{531}.

	**microfacet**	**adsorption state**		
		**intact**	**TS**	**dissociated**
S-Ala	{311}	−1.87	−1.02	−2.37
	{110}	−1.71	-	−2.18
R-Ala	{311}	−1.80	−0.85	−2.28
	{110}	−1.65	-	−2.12

2 Statistical description of MD results

The statistical robustness of the data shown in Figure 4 of the main manuscript was tested as follows:

The null hypothesis that the L_z values of S-Ala are distributed according to the MixtureDistribution[{0.5625, 0.4375}, {NormalDistribution(64.7012, 21.103), NormalDistribution(15.7554, 31.979)}] is not rejected at the 5%-level based on the Cramér-von Mises test, p-value 0.993984

The null hypothesis that the L_z values of R-Ala are distributed according to the MixtureDistribution [{0.209257, 0.317679, 0.473063}, {NormalDistribution(39.0038, 8.78946), NormalDistribution(27.2266, 11.286), UniformDistribution({67.8068, 49.3992})}] is not rejected at the 5%-level based on the Cramér -von Mises test, p-value 0.995534

3 Desorption Velocity of S-Ala and R-Ala from Cu{531}

The desorption velocity shown in Figure 1 was calculated as the change in distance between the centre of mass of the respective alanine molecule and the topmost copper atom of the surface at each time step.

4 MD-Results of R-Ala on Cu{110}

Upon desorption of R-Ala from Cu{110}, the magnitude of the angular momentum vector, L, increases by approx. 70%, with the largest increase between 50 and 150 fs, see the left panel of Figure 2. The mean L_z, shown in the right panel of Figure 2 reaches +21.4 a.u. at the end of the simulation time and thus lies in the same range as seen for S-Ala on R-Cu{531} in the main manuscript. The evolution of the magnitude of the angular momentum vector and its z-component are in agreement with the expected behaviour for a chiral system.[1]

5 Vector depiction of the principal moments of inertia and the angular momentum for R- and S-Ala on Cu{531}

A mean vector of all angular momentum vectors (e.g. the mean of all blue vectors in Figure 3a) was calculated at each of the shown time points. Then the angle between all individual angular momentum vectors and this mean vector was obtained. The

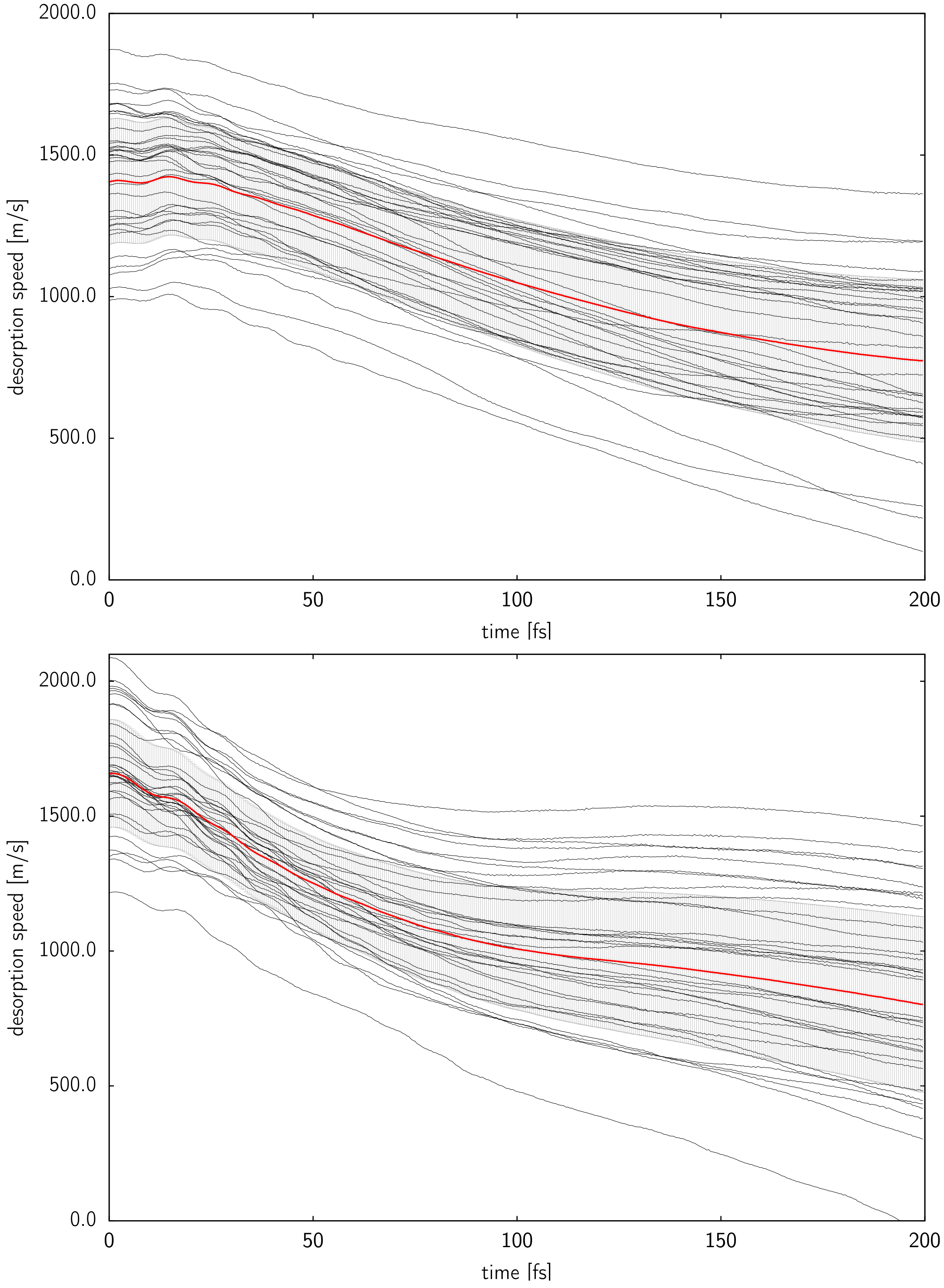

Figure 1: Time evolution of the desorption velocity of (top) S-Ala and (bottom) R-Ala from Cu{531}. Red continuous lines indicate the mean value for the whole ensemble of trajectories desorbing from the surface; an error range of one standard deviation is indicated in grey.

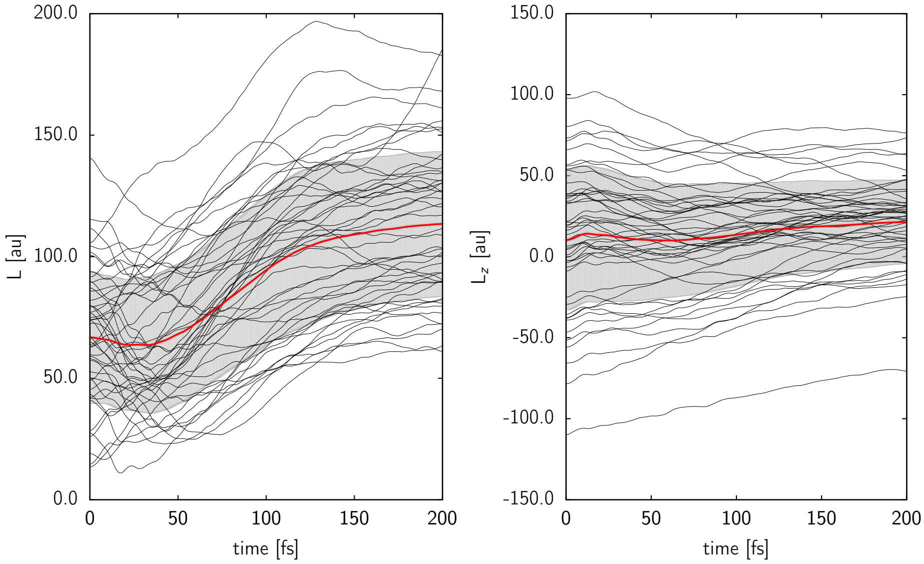

Figure 2: Time evolution of (left) the magnitude of the angular momentum, L of R-Ala and (right) the z-component of the angular momentum vector, L_z of R-Ala on Cu{110}. Red continuous lines indicate the mean value for the whole ensemble of trajectories desorbing from the surface; an error range of one standard deviation is indicated in grey.

average over these angles is the value given in the main manuscript as the "average angle between individual angular momentum vectors and the ensemble mean" and represents a measure of how much the vectors are spread in space, see our previous publication[2] for a more detailed discussion of this approach.

References

[1] S. C. Matysik, D. J. Wales, and S. J. Jenkins. *Phys. Rev. Lett.*, **2021**, 126, *16*, 166101.

[2] S. C. Matysik, D. J. Wales, and S. J. Jenkins. *J. Phys. Chem. C*, **2021**, 125, *51*, 27938–27948.

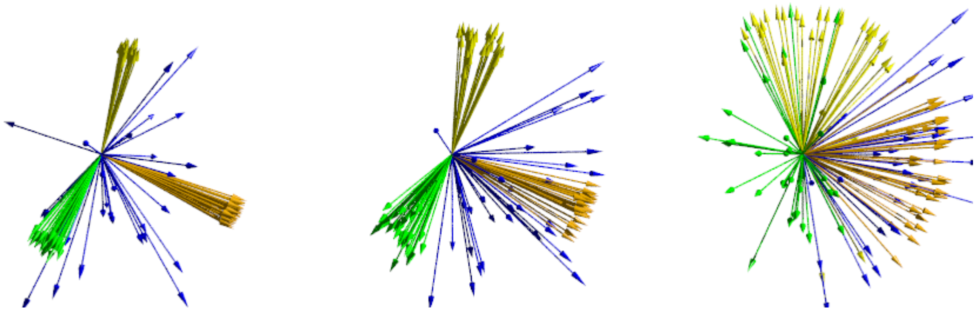

(a) **S-Ala 20 fs** (b) **S-Ala 60 fs** (c) **S-Ala 200 fs**

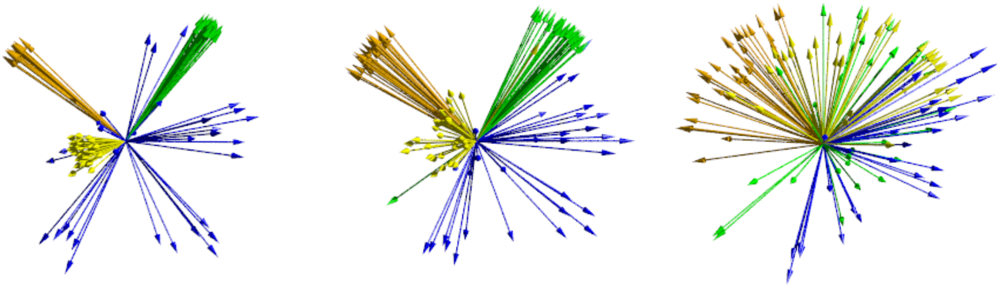

(d) **R-Ala 20 fs** (e) **R-Ala 60 fs** (f) **R-Ala 200 fs**

Figure 3: Time evolution of the angular momentum vector (dark blue) and the principal axes of the inertia tensor (green, yellow, orange) of alanine desorbing from Cu{531} after 20 fs, 60 fs and 200 fs. (Top row) S-Ala (bottom row) R-Ala
